# Correlation Between FDG Hotspots on Pre-radiotherapy PET/CT and Areas of HNSCC Local Relapse: Impact of Treatment Position and Images Registration Method

**DOI:** 10.3389/fmed.2020.00218

**Published:** 2020-06-04

**Authors:** Blandine Truffault, David Bourhis, Anne Chaput, Jeremie Calais, Philippe Robin, Romain Le Pennec, François Lucia, Jean-Christophe Leclère, Dorothy M. Gujral, Pierre Vera, Pierre-Yves Salaün, Ulrike Schick, Ronan Abgral

**Affiliations:** ^1^Department of Nuclear Medicine, Brest University Hospital, Brest, France; ^2^European University of Brittany, Brest, France; ^3^Department of Medical and Molecular Pharmacology, David Geffen School of Medicine, University of California, Los Angeles, Los Angeles, CA, United States; ^4^Department of Nuclear Medicine and Radiology, Henri Becquerel Center, QuantIF (LITIS EA 4108 – FR CNRS 3638), Rouen University Hospital, Rouen, France; ^5^Department of Radiotherapy, Brest University Hospital, Brest, France; ^6^Department of Oto-rhino-laryngology, Brest University Hospital, Brest, France; ^7^Clinical Oncology Department, Imperial College Healthcare NHS Trust, Charing Cross Hospital, Hammersmith, London, United Kingdom; ^8^Department of Cancer and Surgery, Imperial College London, London, United Kingdom

**Keywords:** FDG-PET/CT hotspots, local relapse, HNSCC, images registration, radiotherapy treatment position

## Abstract

**Aim:** Several series have already demonstrated that intratumoral subvolumes with high tracer avidity (hotspots) in 18F-flurodesoxyglucose positron-emission tomography (FDG-PET/CT) are preferential sites of local recurrence (LR) in various solid cancers after radiotherapy (RT), becoming potential targets for dose escalation. However, studies conducted on head and neck squamous cell carcinoma (HNSCC) found only a moderate overlap between pre- and post-treatment subvolumes. A limitation of these studies was that scans were not performed in RT treatment position (TP) and were coregistred using a rigid registration (RR) method. We sought to study (i) the influence of FDG-PET/CT acquisition in TP and (ii) the impact of using an elastic registration (ER) method to improve the localization of hotpots in HNSCC.

**Methods:** Consecutive patients with HNSCC treated by RT between March 2015 and September 2017 who underwent FDG-PET/CT in TP at initial staging (PET_A_) and during follow-up (PET_R_) were prospectively included. We utilized a control group scanned in non treatment position (NTP) from our previous retrospective study. Scans were registered with both RR and ER methods. Various sub-volumes (A_X_; x = 30, 40, 50, 60, 70, 80, and 90%SUVmax) within the initial tumor and in the subsequent LR (R_X_; x = 40 and 70%SUVmax) were overlaid on the initial PET/CT for comparison [Dice, Jaccard, overlap fraction = OF, common volume/baseline volume = A_X_nR_X_/A_X_, common volume/recurrent volume = A_X_nR_X_/R_X_].

**Results:** Of 199 patients included, 43 (21.6%) had LR (TP = 15; NTP = 28). The overlap between A_30_, A_40_, and A_50_ sub-volumes on PET_A_ and the whole metabolic volume of recurrence R_40_ and R_70_ on PET_R_ showed moderate to good agreements (0.41–0.64) with OF and A_X_nR_X_/R_X_ index, regardless of registration method or patient position. Comparison of registration method demonstrated OF and A_X_nR_X_/R_X_ indices (x = 30% to 50%SUVmax) were significantly higher with ER vs. RR in NTP (*p* < 0.03), but not in TP. For patient position, the OF and A_X_nR_X_/R_X_ indices were higher in TP than in NTP when RR was used with a trend toward significance, particularly for x=40%SUVmax (0.50±0.22 vs. 0.31 ± 0.13, *p* = 0.094).

**Conclusion:** Our study suggested that PET/CT acquired in TP improves results in the localization of FDG hotspots in HNSCC. If TP is not possible, using an ER method is significantly more accurate than RR for overlap estimation.

## Introduction

Head and neck squamous cell cancer carcinomas (HNSCC) are the sixth most common cancer (1, 2) with around 800,000 new cases worldwide in 2015. These tumors have a poor prognosis, with a 5-year survival rate <50% (3), particularly because two thirds of patients are unfortunately diagnosed at advanced stage. In addition to surgery, concurrent chemo-radiotherapy is a standard of care in the curative-intent management of locally advanced tumors (4, 5). However, despite improvements in treatment modalities, locoregional failure rates remain high (4, 6).

Several studies have suggested that local recurrence (LR) of HNSCC treated with radiotherapy (RT) occurs mainly within the planning target volume (PTV) regardless of radiotherapy technique, suggesting that the radiation dose delivered may be insufficient for local tumor control (7). RT dose escalation is often limited by the tolerance of surrounding tissues and the associated risk of radiation-induced toxicities (8–10). Therefore, the ability to accurately define and irradiate areas at high risk of recurrence could be useful to guide a boost protocol with the use of modern techniques such as intensity modulated radiotherapy (IMRT) and stereotactic radiotherapy (11, 12).

The usefulness of 18Flurorodesoxyglucose positron emission tomography/computed tomography (FDG-PET/CT) for initial staging, therapeutic assessment and recurrence diagnosis in HNSCC is now well established (13–15). It is also increasingly considered a useful tool in RT to optimize target volume contouring. Indeed, it allows the delineation of target volume boundaries more precisely, with reduction in inter- and intra-observer reproducibility compared to CT (16–19). In addition, FDG-PET/CT is currently being investigated as a tool to guide radiotherapy dose escalation in order to decrease toxicitities and improve tumor control (20). One of the most important studies in this context is the ongoing multicentric trial ARTFORCE (NCT01504815), which compares a standard dose of 70 Gy with an FDG-PET/CT-based simultaneous integrated boost to areas of high FDG uptake (hotspots) up to a maximum dose of 84 Gy (21).

Recent studies have reported a high risk of LR within FDG hotspots identified on pre-RT PET/CT in lung (22–25), rectal (26), and esophageal malignancies (27). Nevertheless, two previous studies conducted on HNSCC failed to confirm good correlation between areas of high FDG uptake and preferential sites of local recurrence (28, 29). Indeed, we recently found only a modest overlap index (<0.6) between pre- and post-treatment subvolumes in 19 recurrent lesions (28). One possible explanation lies in the lack of reproducibility of the patients positioning between the two scans. Moreover, weight loss and post-therapeutic tissues distortion in HNSCC could also affect anatomical landmarks, making the registration process with a rigid approach more difficult.

Our main objective was to prospectively determine if PET-CT acquisition in the same RT position and image co-registration with an elastic registration method could improve the overlap between FDG hotspots and HNSCC local relapse subvolumes. Therefore, the study aimed to investigate whether a difference existed between (i) RR and ER registration methods and (ii) TP versus NTP patient positioning for PET-CT acquisition. We also sought to define the optimal SUVmax threshold to identify the lowest volume on the initial PET that could be used as a reduced target volume of RT.

A secondary objective of this study was to confirm the prognostic value of initial metabolic tumor burden (metabolic tumor volume = MTV and total lesion glycolysis = TLG) in patients with HNSCC.

## Materials and Methods

### Population

Consecutive patients with histologically proven HNSCC treated with RT with or without concomitant systemic treatment referred between March 2015 and September 2017 to our department for FDG-PET/CT were prospectively enrolled in the current study. All patients had FDG-PET/CT before and after treatment in TP. A control group scanned in NTP from our previously published retrospective series was used for comparison (28).

### Treatment Modalities

All patients were treated with RT ± chemotherapy according to international guidelines (30). External RT was delivered using volumetric modulated arc therapy (VMAT) on a Truebeam STx accelerator (Varian®, Palo Alto, USA). The gross tumor volume (GTV) was delineated on a planning CT scan after combining the information provided by endoscopy, contrast-enhanced diagnostic CT or MRI. The dose to the GTV was 70Gy (2Gy/fraction/day, 5 sessions/week) over 7 weeks +/- concomitant systemic treatment: Cetuximab, Cisplatin or Carboplatin.

### Follow-Up

Clinical follow-up was performed as recommended by the National Comprehensive Cancer Network (30). Patients with persistent disease on FDG-PET/CT 3 months after RT completion, and those who, after initial complete response, relapsed within the radiation field during follow-up were pooled together to comprise the LR group. Histological evidence was highly recommended; otherwise evidence of progression on imaging was used to define LR.

The following clinical characteristics were obtained for each patient and considered as variables in univariate analysis: age, sex, tumor location, AJCC stage, systemic treatment modality, RT dose and RT duration.

### FDG-PET/CT Imaging

The first FDG-PET/CT (PET_A_) was performed for initial staging. The post-therapeutic PET_R_ was defined as either the PET performed at the time of the first evaluation (3 months) in patients showing persistant/progressive disease, or the first PET performed during follow-up (suspected recurrence or systematic surveillance) that demonstrated LR. All indications for FDG PET/CT were reviewed according to guidelines (15, 30) by a multidisciplinary team.

All FDG-PET/CT data were acquired on a Biograph-mCT™ system (Siemens®, Erlangen, Germany) in the same institution. The patients were required to fast for at least 6h before imaging. Scans were performed 60 min after injection of ~3–4 MBq/kg of FDG (IBA molecular imaging®, Saclay, France). From March 2015, patients were scanned according to their compliance in TP, supine on a rigid board, neck maintained in a semi-rigid headrest. As previously mentioned, patients from our control group (28) were supine without rigid board or headrest (NTP).

PET data were acquired using a whole-body protocol (2 min per step, 200x200 matrix) and reconstructed using an ordered subsets expectation-maximization (OSEM) algorithm (TrueX™=PSF (point spread function) + time of flight (TOF) OSEM-3D with 4 x 4 x 2 mm voxels. Data were corrected for random coincidences, scatter and attenuation using the CT scan. PET images were smoothed with a Gaussian filter (full-width at half-maximum = 2 mm).

CT data were acquired in the cranio-caudal direction using a whole body protocol. Intravenous iodine contrast agent (1.5 mL/kg) was used for the CT scan unless contra-indicated. The CT consisted of a 64-slice multidetector-row spiral scanner with a transverse field of view of 700 mm. The CT parameters were a collimation of 16 x 1.2 mm, pitch = 1, tube voltage and exposure were automatically regulated (CarekV®, CareDose 4D®) with 120 kV and 80 QrefmAs as basis parameters and CT iterative reconstruction was used (SAFIRE®, strength 5).

The study was approved by our institutional ethics committee (number 2017.CE25). All patients gave written informed consent.

### FDG-PET/CT Analysis

All scans were analyzed by the same nuclear medicine physician (BT). The registration and overlap comparisons were then performed using the MIM™ software (MIM™ Software Inc., Cleveland, USA). For each patient, two registration methods (RR and ER) were studied.

For the RR method, the CT of PET_A_ (CT_A_) was registred with CT of PET_R_ (CT_R_), focusing on the tumor area. Regions of interest were identified and outlined on the CT, using PET images as reference. Manual adjustment was not allowed. The transformations derived from the CT registration process were then reported on PET_A_ images.

The ER method was performed by the VoxAlign® Engine algorithm, a constrained intensity based, free-form registration (31). A rigid registration between CT_A_ and CT_R_ was first performed, followed by ER. The deformable registration matrix was saved, and applied to PET_A_. These deformations led to an elastic registered CT and PET.

**Figure 1:**
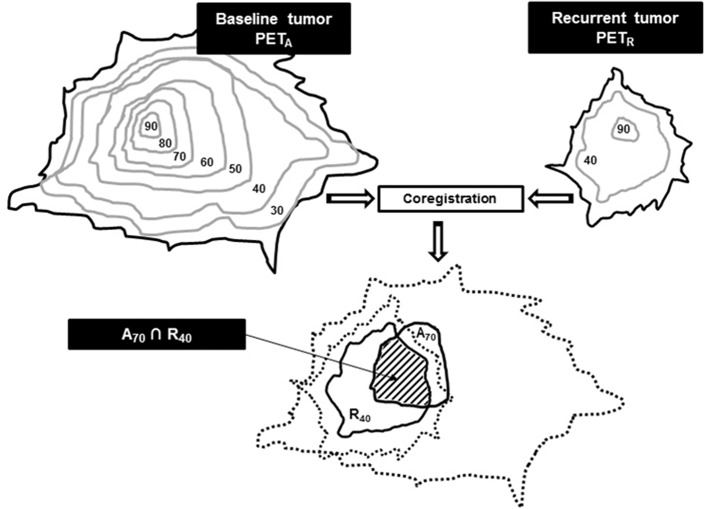
Typical A_70_ and R_40_ sub-volumes overlap estimation after co-registration and reports. Use with permission of Calais et al. (27).

Seven volumes of interest (VOIs) on PET_A_ and 2 VOIs on PET_R_ (metabolic active residual disease or relapse) were respectively defined. On PET_A_, baseline sub-volumes were delineated using a relative threshold method (A_X_ with x = 30, 40, 50, 60, 70, 80, and 90% of SUVmax). On PET_R_, thresholds at x = 40 and 70% of SUVmax were respectively used to delineate R_40_ and R_70_ recurrent sub-volumes. Baseline sub-volumes A_X_ were reported on PET_R_, and recurrence sub-volumes R_X_ were reported on PET_A_, to quantify their respective overlaps (Figure 1).

The following quantitative parameters were also collected on PET_A_ for the prognosis analysis: SUVmax, SUVmean, MTV, and TLG (TLG= SUVmean × MTV).

### Overlap Estimation

All potential overlaps between baseline tumor sub-volumes (A_30_ to A_90_) vs. relapse (R_40_ and R_70_) sub-volumes were investigated using five indices [Dice, Jaccard, overlap fraction (OF), common volume divided by the initial volume (A_X_nR_X_/A_X_) and common volume divided by the compared volume (A_X_nR_X_/R_X_)], as recommended by Calais et al. (22, 27) and as applied in our previous study (28).

Index values for each parameter vary between 0 if the volumes are completely disjointed and 1 if the volumes match perfectly in size, shape and location.

This overlap analysis was conducted on 4 subgroups: NTP-RR, NTP-ER, TP-RR, and TP-ER.

**Figure 2:**
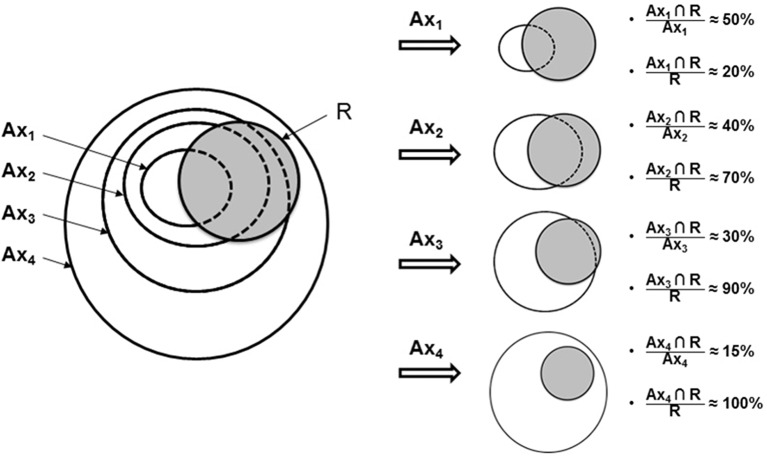
Study flow for scenario of PET_A_ and PET_R_ sub-volume comparisons. Indices of common volume (A∩R) with A referring to baseline PET and R to PET at recurrence. Use with permission of Calais et al. (27).

A schematic example of the interpretation of overlap indices is represented in Figure 2.

### Statistics

The quality of overlap was assessed using Cohen k-test for agreement between investigators as follows: 0–0.2, poor agreement; 0.21–0.40, fair agreement; 0.41–0.60, moderate agreement; 0.61–0.80, good agreement; and 0.81–1.00, very good agreement (32). Comparison of mean overlap in different subgroups was performed using the Wilcoxon or Mann-Whitney *U*-tests as appropriate. The statistical associations between FDG-PET/CT and clinical parameters were tested using a repeated measures analysis of variances (ANOVA) and the chi-2 squared test. A *p* < 0.05 was considered statistically significant. All analyses were performed using XLSTAT (Addinsoft®, Paris, France).

## Results

### Patients Characteristics

The final cohort included 199 patients (142M/57F). Characteristics of patients are reported in Table 1.

**Table 1:** Characteristics of the 199 patients included in the study.

	**TOTAL**	**CR**	**DR**	**LR**
	**(*n* = 199)**	**(*n* = 120)**	**(*n* = 36)**	**(*n* = 43)**
Age, yo ± SD	62.5 ± 10.2	61.3 ± 8.9	64.0 ± 10.2	64.7 ± 12.7
Gender, *n* (%)				
Male	142(71.4)	85(70.8)	26(72.2)	31(72.1)
Female	57(28.6)	35(29.2)	10(27.8)	12(27.9)
Tumor location, *n* (%)				
Rhinopharynx	7(3.5)	5(4.2)	1(2.8)^*^	1(2.3)^*^
Oropharynx	124(62.3)	70(58.3)	26(72.2)^*^	28(65.1)^*^
Hypopharynx	24(12.1)	14(11.7)	5(13.9)^*^	5(11.6)^*^
Larynx	31(15.6)	26(21.7)	3(8.3)^*^	2(4.7)^*^
Oral cavity	13(6.5)	5(4.2)	1(2.8)^*^	7(16.3)^*^
AJCC stage, *n* (%)				
I	5(2.5)	5(4.2)	0^*^	0^*^
II	16(8.0)	14(11.7)	0^*^	2(4.6)^*^
III	40(20.1)	28(23.3)	5(13.9)^*^	7(16.3)^*^
IV	138(69.3)	73(60.8)	31(86.1)^*^	34(79.1)^*^
RT Duration, (days ± SD)	54.8 ± 9.1	53.4 ± 9.4	56.7 ± 7.5	57.3 ± 8.5
RT Dose, (Gy ± SD)	70.0 ± 1.1	70.0 ± 0.5	70.3 ± 1.2	69.9 ± 1.8
Treatment, *n* (%)				
CRT	137(68.9)	85(20.8)	26(72.2)	26 (60.5)
Single RT	56(28.1)	31(25.8)	8(22.2)	17(39.5)

*CR, Complete response; DR, Distant relapse; LR, Local relapse; SD, Standard deviation; CRT, Chemo-radiotherapy*;

* *Significantly different fromCR*.

Of these, 137 (68.8%) received concomitant systemic treatment: Cetuximab (20/199), Cisplatin or Carboplatin ± 5-FU (117/199).

**Figure 3:**
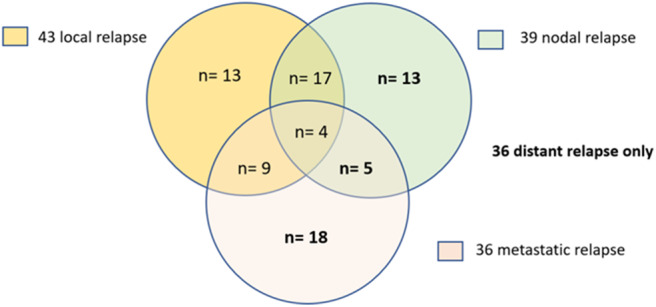
Patterns of relapse. LR (local relapse) and DR (distant relapse).

The mean ± SD time of follow-up of the population was 18.7 ± 11.3 months. At last follow-up, 120 (60.3%) with initial complete response remained free of disease (CR) and 43 (21.6%) experienced local relapse (LR). Twenty-nine LR were identified on imaging and confirmed pathologically. Fourteen LR were considered as such based on evidence of local and metastatic progression disease on any imaging procedure or on clinical examination. Thirty-six patients (18.1%) showed distant dissemination (nodal or metastatic) without LR (Figure 3).

### PET/CT Parameters

Fifteen patients with LR were scanned in TP and 28 in NTP.

The initial PET/CT parameters are summarized in Table 2. The mean ± SD initial MTV was 7.7 ± 7.8 cc in the entire cohort and 9.1 ± 7.2 cc in patients with LR, wheras mean ± SD initial TLG was 90.6 ± 101.8 g and 105.9 ± 80.6 g, respectively.

**Table 2:** Metabolic parameters on baseline PET/CT.

	**TOTAL (*n* = 199)**	**CR (*n* = 120)**	**DR (*n* = 36)**	**LR (*n* = 43)**	**GR (*n* = 79)**
SUVmax	20.0 ± 9.6	19.2 ± 9.5	20.1 ± 6.1	21.2 ± 8.2	20.7 ± 7.3
SUVmean	12.0 ± 5.6	11.3 ± 5.7	12.3 ± 3.8	12.5 ± 4.8	12.4 ± 4.3
MTV (cc)	7.7 ± 7.8	6.4 ± 7.1	9.8 ± 8.4^*^	9.1 ± 7.2^*^	9.4 ± 7.7^*^
TLG (g)	90.6 ± 101.8	75.2 ± 98.4	120.7 ± 111.4^*^	105.9 ± 80.6^*^	112.7 ± 95.5^*^

* *Significantly different from CR. MTV = A_40_*.

### Overlap Comparison

**Figure 4:**
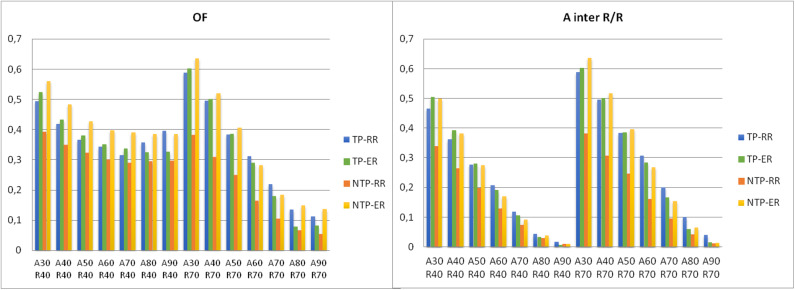
Histogram of the mean values of OF and AxnRx/Rx index for various SUVmax thresholds to delineate the volumes on PET_A_ (baseline) and PET_R_ at relapse in the 4 subgroups.

A total of 6,020 overlap indices were obtained, i.e., 140 potential overlaps between the baseline PET_A_ VOIs and relapse PET_R_ VOIs in the 43 patients who had LR and using the 2 registration methods (387 VOIs on 86 PET/CT). Two typical examples are shown in Figure 4. Mean overlap index values are reported in Table 3.

**Table 3:** Mean values of overlap indices for various SUVmax thresholds to delineate volumes on PET_A_ baseline (A_X_) and PET_R_ at relapse (R_40_ and R_70_).

				**R_**40**_**							**R_**70**_**			
	**A_**30**_**	**A_**40**_**	**A_**50**_**	**A_**60**_**	**A_**70**_**	**A_**80**_**	**A_**90**_**	**A_**30**_**	**A_**40**_**	**A_**50**_**	**A_**60**_**	**A_**70**_**	**A_**80**_**	**A_**90**_**
**DICE**														
*RR TP*	0.25	0.24	0.22	0.20	0.15	0.07	0.03	0.08	0.09	0.10	0.10	0.10	0.08	0.05
*ER TP*	0.25	0.25	0.22	0.18	0.13	0.06	0.01	0.06	0.07	0.08	0.09	0.08	0.06	0.02
*RR NTP*	0.22	0.21	0.19	0.15	0.10	0.05	0.02	0.06	0.06	0.07	0.06	0.06	0.04	0.02
*ER NTP*	0.32	0.30	0.26	0.19	0.13	0.06	0.02	0.10	0.11	0.12	0.12	0.10	0.07	0.02
**Jacquard**														
*RR TP*	0.15	0.15	0.14	0.12	0,09	0.04	0.02	0.05	0.05	0.06	0.06	0.05	0.05	0.03
*ER TP*	0.15	0.15	0.13	0,11	0.08	0.03	0.01	0.03	0.04	0.04	0.05	0.04	0.03	0.01
*RR NTP*	0.13	0.13	0.11	0.09	0.06	0.03	0.01	0.03	0.03	0.04	0.03	0.03	0.02	0.01
*ER NTP*	0.20	0.19	0.16	0.12	0.07	0.03	0.01	0.05	0.06	0.07	0.07	0.06	0.04	0.01
**OF**														
*RR TP*	**0.49**	**0.42**	0.37	0.34	0.31	0.36	0.40	**0.59**	**0.50**	0.38	0.31	0.22	0.14	0.11
*ER TP*	**0.52**	**0.43**	0.38	0.35	0.34	0.32	0.33	**0.61**	**0.50**	0.39	0.29	0.18	0.08	0.08
*RR NTP*	0.39	0.35	0.32	0.30	0.29	0.29	0.30	0.38	0.31	0.25	0.16	0.10	0.06	0.05
*ER NTP*	0.56^*^	0.48^*^	0.43^*^	0.40	0.39	0.39	0.38	0.64^*^	0.52^*^	0.41^*^	0.28	0.19	0.15	0.14
**A∩R/A**														
*RR TP*	0.21	0.23	0.26	0.29	0.31	0.36	0.40	0.05	0.06	0.07	0.07	0.08	0.10	0.11
*ER TP*	0.20	0.24	0.27	0.30	0.33	0.32	0.33	0.03	0.04	0.05	0.06	0.07	0.07	0.08
*RR NTP*	0.22	0.24	0.27	0.28	0.29	0.29	0.30	0.04	0.04	0.04	0.04	0.05	0.06	0.05
*ER NTP*	0.30	0.34	0.36	0.37	0.39	0.39	0.38	0.06	0.07	0.08	0.10	0.12	0.13	0.14
**A∩R/R**														
*RR TP*	**0.47**	0.36	0.28	0.21	0.12	0.04	0.02	**0.59**	**0.50**	0.38	0.31	0.20	0.10	0.04
*ER TP*	**0.50**	0.39	0.28	0.19	0.11	0.03	0.01	**0.61**	**0.50**	0.39	0.28	0.17	0.06	0.01
*RR NTP*	0.34	0.26	0.20	0.13	0.07	0.03	0.01	0.38	0.31	0.25	0.16	0.10	0.04	0.01
*ER NTP*	**0.50**	0.38	0.27	0.17	0.09	0.04	0.01	0.64^*^	0.52^*^	0.40	0.27	0.15	0.06	0.01

*See the text for a description of the indices*.

Highest mean values in bold (moderate to good agreement), significantly different from RR NTP (

* ).

#### Ax vs. R_40_ Comparisons

None of the indices showed good or very good agreement. The OF(A_X_nR_40_) index showed moderate agreement (0.42–0.56) for SUVmax thresholds of 30–40% in TP-RR, TP-ER, and NTP-ER subgroups. The OF(A_50_nR_40_) index showed moderate agreement (0.43) in NTP-ER subgroup. The A_30_nR_40_/R_40_ index showed moderate agreement (0.47–0.50) in TP-RR, TP-ER, and NTP-ER subgroups.

The Dice, Jaccard and A_X_nR_40_/A_X_ indices showed poor to fair agreement.

#### Ax vs. R_70_ Comparisons

None of the indices showed very good agreement. The OF(A_X_nR_70_) and A_X_nR_70_/R_70_ indices showed moderate to good agreement (0.50–0.64) for SUVmax thresholds of 30–40% in the TP-RR, TP-ER and NTP-ER subgroups. The OF(A_50_nR_70_) index showed moderate agreement (0.41) in the NTP-ER subgroup.

The Dice, Jaccard and A_X_nR_70_/A_X_ indices were very low, mostly below 0.20, irrespective of the thresholds used on PET_A_.

#### TP vs. NTP

With RR method, the OF(A_X_nR_70_), A_X_nR_40_/R_40_, and A_X_nR_70_/R_70_ indices for SUVmax thresholds of 30–40% were higher in TP subgroup than in the NTP subgroup with a trend toward significance. For example, the OF(A_30_nR_70_) and A_30_nR_70_/R_70_ indices were higher in the TP subgroup (0.59 ± 0.22) than in the NTP (0.38 ± 0.14) subgroup (*p* = 0.10); and OF(A_40_nR_70_) and A_40_nR_70_/R_70_ were higher in the TP subgroup (0.50 ± 0.22) than in the NTP (0.31 ± 0.13) subgroup (*p* = 0.094).

With ER method, there was no significant difference between TP and NTP subgroups with the aboved-mentioned best agreement (moderate to good) of OF(A_X_nR_40_), A_X_nR_40_/R_40_, and A_X_nR_70_/R_70_ indices.

#### Elastic vs. Rigid Registration Method

**Figure 5:**
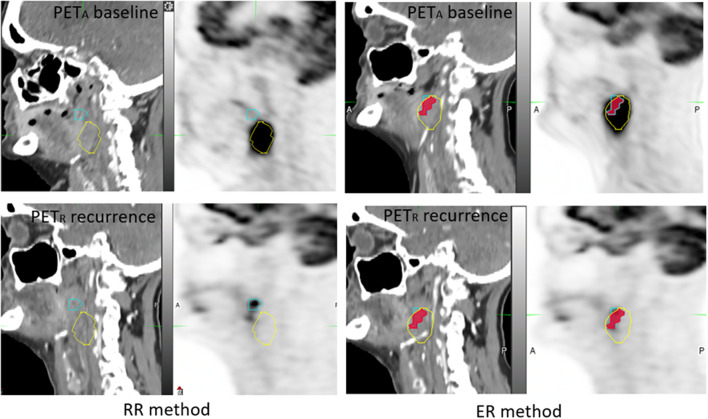
Example of 65 year-old woman, with T3N1M0 oropharyngeal squamous cell carcinoma. Before the emergence of reflex otalgia and neck pain, a PET-CT was performed 2 months after radiotherapy and showed a local persistant disease. PET_A_ (images on the top) and PET_R_ (images on the bottom) were not scanned in treatment position (NTP) and coregistered with RR method (on the left) and ER method (on the right). A_40_ subvolume (yellow line), R_40_ subvolume (blue line) and A_40_∩R_40_ (red area). The OF (A_40_, R_40_) index was calculated respectively at 0 and 0.80 for RR and ER registration methods.

In the NTP subgroup, the OF(A_X_nR_40_) and OF(A_X_nR_70_) indices for SUVmax thresholds of 30–50% were significantly higher with ER than those obtained using the RR method (*p* < 0.03). The A_X_nR_70_/R_70_ index values for SUVmax thresholds of 30–40% were significantly higher with ER vs. RR method (*p* = 0.028). A typical example is shown in Figure 5.

**Figure 6:**
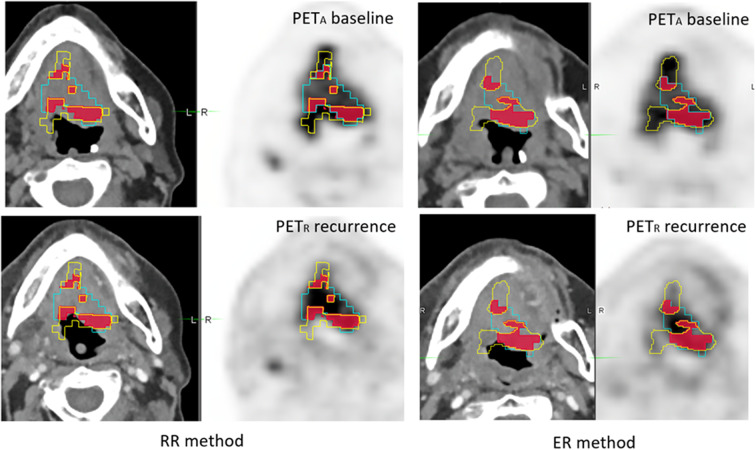
Example of 56 year-old woman with T4N2M0 oropharyngeal squamous cell carcinoma. A therapeutic response assessment by PET/CT was performed 3 months after chemo-radiotherapy, showing persistent disease. PET_A_ (images on the top) and PET_R_ (images on the bottom) were scanned in treatment position (TP) and coregistered with RR method (left) and ER method (right). A_40_ subvolume is represented as a yellow line, R_40_ subvolume as a blue line and A_40_∩R_40_ in red area. The OF (A_40_, R_40_) index was calculated, respectively at 0.50 and 0.51 for RR and ER registration methods.

**Figure 7:**
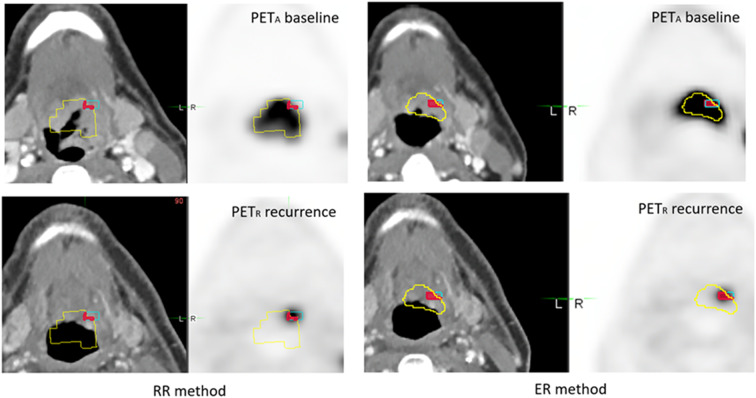
Example of 49 year-old man with an T4N2M0 oropharyngeal HNSC. During routine clinical monitoring, a FDG-PET/CT scan was performed 7 months after chemoradiotherapy, and demonstrated an occult local relapse. PET_A_ (images on the top) and PET_R_ (images on the bottom) were scanned in treatment position (TP) and coregistered with RR method (left) and ER method (right). A_40_ subvolume is represented as a yellow line, R_40_ subvolume as a blue line and A_40_∩R_40_ in red area. The OF (A_40_, R_40_) index was calculated respectively at 0.19 and 0.64 for RR and ER method.

In the TP subgroup, there was no significant difference between the RR and ER methods neither with the aboved-mentioned best agreement (moderate to good) of the OF(A_X_nR_X_), A_X_nR_40_/R_40_ and A_X_nR_70_/R_70_ indices (Figure 6), nor regarding the lowest. There was only one case where the overlaps increased by a factor 3 (Figure 7).

### Univariate Analysis

Gender, RT dose, RT duration, use of chemotherapy, baseline SUVmax and SUVmean were not statistically different between patients with complete response (CR), distant relapse (DR) or local relapse (LR). However, patients with CR were significantly younger (*p* = 0.021), and more often presented with early stage disease (*p* = 0.003), and laryngeal cancer (larynx, *p* = 0.027).

The mean ± SD MTV on baseline PET was significantly lower in the controlled patients (6.4 ± 7.1 cc) than all relapsed patients (sum of DR+LR) (9.4 ± 7.7 cc, *p* = 0.006), DR patients (9.8 ± 8.4 cc, *p* = 0.031), and LR patients (9.1 ± 7.2 cc, *p* = 0.041), The mean±SD TLG on baseline PET was significantly lower in controlled patients (75.2 ± 98.4 g) than all relapsed patients (112.7 ± 95.5 g, *p* = 0.008) DR patients (120.7±111.4 g, *p* = 0.032), and LR patients (105.9 ± 80.6g, *p* = 0.046),

## Discussion

The rationale for applying the “hotspot” localization concept to HNSCC relies on the overlap of the recurrence sites with the pre-RT biological target volume (BTV). Indeed, Soto et al. reported that LR was included in the pre-treatment FDG BTV in 8/9 patients after RT (33). Based on these findings, recent articles have suggested the use of biological and functional parameters in FDG-PET/CT to identify the radioresistant tumor area (14, 34). Thereby, Jeong and al. suggested that FDG-avid tumors require at least 10–30% higher dose than non-FDG avid tumors (14).

Whilst studies on lung (22–25), rectal (26), and esophageal (27) cancers have shown good to excellent agreements between intra-tumoral FDG hotspots and areas of local recurrence, disappointing results have been obtained in series conducted on HNSCC. Indeed, Chaput et al. reported a moderate correlation between volumes identified on initial PET_A_ and relapse PET_R_. The authors demonstrated OF(A_X_nR_40_) and AxnR_40_/R_40_ indices showed a moderate agreement (0.52–0.43) for SUVmax thresholds of 30–50%. Moreover, moderate agreement values (0.54–0.45) of OF(A_X_nR_70_) and A_X_nR_70_/R_70_ indices were obtained for baseline SUVmax thresholds between 30 and 40% (28). Using a similar PET procedure (no treatment position, rigid registration), Legot et al. reported comparable results in 38 patients: the OF(A_X_nR_40_) ranged between 0.35 and 0.55 for overlaps between R_40_ and A_30_, A_40_, A_50_, and A_60_, respectively. Similarly, AxnR_X_/R_X_ showed only a fair overlap with values ranging between 0.3 and 0.4 for comparison of R_40_ with A_40_, A_50_, A_60_, A_70_, and A_80_ (29).

Therefore, we hypothesized that performing PETs in TP as well as using deformable registration could improve the methodogy of this process and translate into better results. Our current study is the first aimed at identifying tumor areas of high risk of relapse in HNSCC using PET-CT images acquired in TP and registered with an ER method. This current work relies on 5 overlap indices with 2 registration methods on 43 HNSCC patients with local failures.

This study confirmed our first hypothesis that patient positioning remains essential, with improved overlap between LR and initial FDG tumor hotspots subvolumes in patients scanned with radiotherapy head support. In comparison to the control group (28), we noted the best agreement (moderate to good) of OF(A_X_nR_70_) and A_X_nR_70_/R_70_ indices for SUVmax threshold of 30 and 40% in the TP group, ranging from 0.50 to 0.61. For example, the OF(A_40_nR_70_) and A_40_nR_70_/R_70_ index values were 0.50 ± 0.22 in the TP group vs. 0.31 ± 0.13 in the NTP group (*p* = 0.094). Admittedly, we have only shown a trend toward significance; however, the lack of statistical power is probably due to a small number of patients in the TP group (15 vs. 28 in the NTP group).

We have also demonstrated that using an elastic method is preferable for image registration when patients cannot be scanned in TP. The OF(A_X_nR_40_), OF(A_X_nR_70_), and A_X_nR_70_/R_70_ index values were significantly better with the ER method for SUVmax threshold of 30–50%. For instance, the OF(A_30_nR_70_) index value was good (0.64 ± 0.15) with ER vs. moderate (0.38 ± 0.14) with RR (*p* = 0.014). To our knowledge, only two other studies have been conducted using elastic image registration software. The first, conducted by Due and al. (35), on a cohort of 39 HNSCC after IMRT. However, for this study the PET baseline volumes were delineated based on visual assessment without an SUV-based semi-automated method, therefore increasing the risk of inter and intra-observer variability. Furthermore, the authors determined the overlap between subvolumes segmented on a PET_BASELINE_ and a CT_RECURRENCEr_, but not on a PET_RECURRENCE_. Shusharina et al. prospectively studied 19 post-RT residual disease of non squamous cell lung carcinoma (NSCLC) and reported the overlap fraction of an initial sub-volume defined as the 50%SUVmax threshold and a relapse sub-volume defined as the 80%SUVmax threshold. They showed that the obtained OF(A_50_nR_80_) was excellent (80%) at 2 weeks after treatment and remained good (63%) at 3 months (25).

Nevertheless, despite RT position and ER method, the hotspot on pre-RT PET-CT that is used to guide definition of areas of high risk of recurrence in patients with HSNCC remains large, and would result in a risk of error with regards to dose escalation. Indeed, the only SUVmax threshold to reach a good agreement value was 30%, which is significantly lower than the threshold obtained in previous studies conducted on other primary tumors. Aerts et al. suggested a 50%SUVmax threshold for delineation on PET_A_ following the OF(A_50_nR_90_) values higher than 70% obtained in their retrospective analysis on 22 patients with local recurrences of NSCLC (23). However, Calais et al. reported that the baseline PET subvolume defined by the 70% SUVmax threshold was an acceptable choice for dose escalation in lung cancer. This choice of threshold could prevent missing the hotspot of recurrence (A_70_nR_90_/R_90_ and OF(A_70_nR_90_) index > 51%) and limit the irradiation of areas at low risk of relapse (A_70_nR_40_/A_70_ and OF(A_70_nR_40_) index > 70%) (22). With this hypothesis, investigators recently assessed the feasibility of FDG PET-guided dose escalation with IMRT in 21 patients with lung cancer. With a boost to A_70_ FDG hotspot, the mean dose to planning target volume was 72.5 ± 0.25 Gy and the dose/volume (D/V) constraints to organs at risk (OAR) were respected (36). Finally, Calais et al. reported good agreement in OF(A_X_nR_40_) for threshold of 30–60% in the 35 patients with LR of esophageal cancer. Likewise, good to excellent OF(A_X_nR_90_) values (0.61–0.89) for threshold of 30 to 60% were reported. The authors also recommended a 60% SUVmax threshold on PET_A_ to delineate high FDG uptake areas on pre-RT PET/CT for a dose escalation target volume (27).

Two main hypotheses could explain why overlap index values in HNSCC are lower than in lung and esophageal cancers. Firstly, although HNSCC are frequently locally advanced at diagnosis, we noticed that MTV values were smaller in our HNSCC series (9.1 ± 7.2cc) than those reported in esophageal (25.4 ± 16.2cc) or lung (53.7 ± 45.6cc) cancers, when considering the same SUVmax threshold of 40% (22, 27). Unlike these above-mentioned tumors, head and neck cancers are known to include necrotic areas without any metabolic activity so the MTV is smaller than real tumor volume. Consequently, with smaller MTV, any mismatch during the registration process can lead to a greater overlap error. Second, weight loss and post-therapeutic tissue distortions are probably more important in HNSCC, with displacement of anatomical landmarks and rendering the registration process more difficult, even with an elastic method.

For our secondary objective, we confirmed that initial MTV and TLG on baseline PET were significantly higher in relapsed patients than locally controlled patients (*p* = 0.041 and *p* = 0.046 respectively) and appeared to be a better prognostic marker than SUVmax (*p* > 0.05). These results are also consistent with previous studies (37, 38). Mapelli et al. studied the value of MTV and TLG to predict outcomes in oropharyngeal carcinomas treated by tomotherapy with simultaneaous integrated boost in FDG-avid tumor subvolumes. They demonstrated that MTV > 4.4cc and TLG > 34.6g were associated with a better 3-year overall survival (*p* = 0.006 and *p* = 0.01, respectively) in a series of 41 patients (39). These results are concordant with our findings.

Textural analysis on pre-RT FDG-PET/CT, already recognized as a prognostic factor for survival (40), could be an interesting approach to predict HNSCC local recurrence sites. Beaumont et al. showed that 15 parameters extracted from a voxel to voxel analysis, combining radiomics and spatial location, allowed better prediction of local failure than a regional analysis, with a median area under the receiver-operating curve of 0.71 (41). The published literature to date mainly underlines methods in assessing tumor hypoxia, a well-known factor for RT resistance (42, 43). Thureau et al. reported that IMRT dose-painting with pre-RT 18F-misonidazole (F-MISO) PET/CT provided NSCLC radiotherapy plan matching with dose/volume (D/V) objectives and organs at risk (OAR) tolerance (36). Patients with F-MISO positive scans who received an RT boost (70 to 86Gy) tend to have a better overall survival (median 26.5 vs. 15.3 months, *p* = 0.71) (44).

Our study has some limitations. First, although larger than previous series, the number of included patients (43 LR) is relatively low, contributing to the lack of power and the inability to confirm superiority of TP when the RR method was used (28, 29). With regards to the results on the prognostic performance of the PET parameters, despite the inclusion of 199 patients, we acknowledge that the role of FDG-PET/CT in systematic follow-up to diagnose occult relapse is still not well defined, despite high performance (45, 46). Our population lacked homogeneity, with a higher proportion of patients with younger age, AJJC I-II stages, and laryngeal cancers included in the CR group. These variables are correlated with lower risk of LR (47, 48). In addition, relapse in FDG-avid lymph nodes at initial staging was not considered. It would be of interest to test the technical feasibility of this process on involved nodes, as these may also benefit for dose escalation, particularly in N3 disease. Finally, we used a PET segmentation method based on different relative SUVmax thresholds. This procedure remains a simple measurement that is easy to perform using commercially available software tools and was utilized in many studies. Van den Bogaard et al. are the only group to utilize an adaptative threshold method based on signal-to-background (26), and reported additional value compared to cancer clinical characterization alone (18, 49). However, this technique remains more tedious to implement and requires a PET calibration phase. Combinations of thresholds could lead to over- or under-estimation of overlaps, and other PET segmentation methods, like automatic approaches should also be tested in future. In fact, several studies have suggested that the gradient-based method (50) best estimates the true tumor volume in NSCLC or HNSCC compared to the SUV-based method (51, 52). Moreover, the segmentation using the FLAB algorithm (fuzzy locally adaptive Bayesian) (53) is also an interesting model that may improve MTV delineation (54, 55). Unfortunately, this patented method is not freely available.

## Conclusion

This study suggests that treatment position improves correlation between FDG hotspot areas on pre-RT PET/CT and sites of local relapse on post-RT PET/CT. When PET in TP is not possible, the use of an elastic registration method is significantly more accurate than a rigid registration method for overlap estimation. However, we found lower overlap index values (at best moderate to good agreement, with SUVmax thresholds of 30–50%) than those reported in other cancers. Further larger prospective studies are needed to assess other PET segmentation methods.

## Data Availability Statement

The datasets generated for this study are available on request to the corresponding author.

## Ethics Statement

The studies involving human participants were reviewed and approved by CHRU Brest institutional ethic committee (n2017.CE25). The patients/participants provided their written informed consent to participate in this study. Written informed consent was obtained from the individual(s) for the publication of any potentially identifiable images or data included in this article.

## Author Contributions

BT, P-YS, and RA are the guarantors of the paper. BT, AC, JC, PV, P-YS, and RA designed the study. US, FL, and J-CL ensured inclusion and follow-up of patients. DB managed imaging procedures. BT, RL, PR, and RA analyzed the data. BT, DB, and RA, realized statistics. DG reviewed the English language. All authors contributed in drawing up the manuscript.

## Conflict of Interest

The authors declare that the research was conducted in the absence of any commercial or financial relationships that could be construed as a potential conflict of interest.
